# Supplementary material for: Fast and High-Efficiency Synthesis of Capsanthin in Pepper by Transient Expression of Geminivirus
Source: Int J Mol Sci. 2023 Oct 9;24(19):15008. doi: 10.3390/ijms241915008 (PMC10573693; doi:10.3390/ijms241915008)
Supplement: Supplementary file 1 [file ijms-24-15008-s001.zip › ijms-2629362-supplementary.pdf]

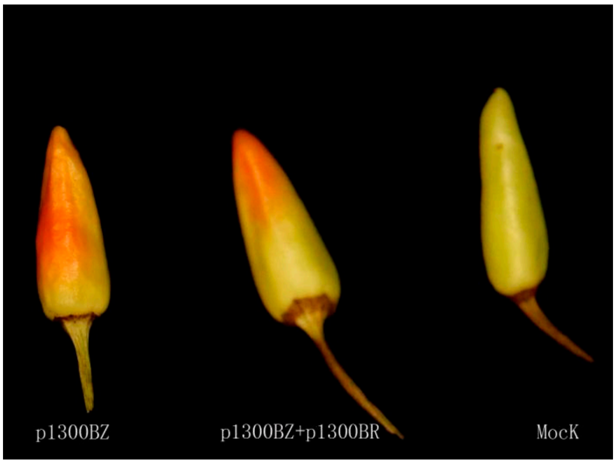

Figure S1. The phenotypic observation of p1300BZ，p1300BZ+p1300BR, with Mock as a control.

Table S1. Amino acid and DNA sequences of studied genes and promoters.

**Name**	**Sequence**
CrtW gene (Amino acid sequence)	MTAAVAEPRIVPRQTWIGLTLAGMIVAGWGSLHVYGVYFHRWGTSS LVIVPAIVAVQTWLSVGLFIVAHDAMHGSLAPGRPRLNAAVGRLTL GLYAGFRFDRLKTAHHAHHAAPGTADDPDFYAPAPRAFLPWFLNF FRTYFGWREMAVLTALVLIALFGLGARPANLLTFWAAPALLSALQLF TFGTWLPHRHTDQPFADAHHARSSGYGPVLSLLTCFHFGRHHEHHL TPWRPWWRLWRGES
CrtW gene (Optimized sequence)	ATGGCTTGGTTAACATGGATTGCATTGTTTCTTACTGCATTTCTTG GAATGGAAGCATTTGCTTGGATTATGCATAGATATGTGATGCAT GGATTTCTTTGGAGTTGGCATAGGTCACATCATGAACCACATGAT CATCCTTTAGAAAAAAATGATTTATTTGCTGTTATTTTTGCTGCTC CAGCAATTGTTATGGTGGCAGTGGGACTTCATCTTTGGCCTTGGG CACTTCCAGTGGGTTTAGGTATTACAGCTTATGGTATGGTGTATT TTTTTTTTCATGATGGACTTGTGCATAGGAGGTTTCCTACAGGATT TAGTGGTAGATCAGGATTTTGGACAAGGCGTATACAAGCACATA GGCTTCATCATGCAGTGAGAACTAGAGAAGGTTGTGTGTCATTT GGTTTTCTTTGGGTGAGGTCTGCTAGAGCACTTGAAGCTGAATTA GCTCAAAAAAGGGGAAGTAGTAGTTCAGGAGCTTAATGA
CrtZ gene (Amino acid sequence)	MAWLTWIALFLTAFLGMEAFAWIMHRYVMHGFLWSWHRSHHEPH DHPLEKNDLFAVIFAAPAIVMVAVGLHLWPWALPVGLGITAYGMVYF FFHDGLVHRRFPTGFSGRSGFWTRRIQAHRLHHAVRTREGCVSFGFL WVRSARALEAELAQKRGSSSSGA
CrtZ gene (Optimized sequence)	ATGGCTTGGTTAACATGGATTGCATTGTTTCTTACTGCATTTCTTG GAATGGAAGCATTTGCTTGGATTATGCATAGATATGTGATGCAT GGATTTCTTTGGAGTTGGCATAGGTCACATCATGAACCACATGAT CATCCTTTAGAAAAAAATGATTTATTTGCTGTTATTTTTGCTGCTC

	CAGCAATTGTTATGGTGGCAGTGGGACTTCATCTTTGGCCTTGGG<br>CACTTCCAGTGGGTTTAGGTATTACAGCTTATGGTATGGTGTATT<br>TTTTTTTTCATGATGGACTTGTGCATAGGAGGTTTCCTACAGGATT<br>TAGTGGTAGATCAGGATTTTGGACAAGGCGTATACAAGCACATA<br>GGCTTCATCATGCAGTGAGAACTAGAGAAGGTTGTGTGTCATTT<br>GGTTTTCTTTGGGTGAGGTCTGCTAGAGCACTTGAAGCTGAATTA<br>GCTCAAAAAAGGGGAAGTAGTAGTTCAGGAGCTTAATGA
35 S promoter sequence	TGAGACTTTTCAACAAAGGGTAATATCCGGAAACCTCCTCGGAT<br>TCCATTGCCCAGCTATCTGTCACTTTATTGTGAAGATAGTGGAAA<br>AGGAAGGTGGCTCCTACAAATGCCATCATTGCGATAAAGGAAA<br>GGCCATCGTTGAAGATGCCTCTGCCGACAGTGGTCCCAAAGATG<br>GACCCCCACCCACGAGGAGCATCGTGGAAAAAGAAGACGTTCC<br>AACCACGTCTTCAAAGCAAGTGGATTGATGTGATATCTCCACTG<br>ACGTAAGGGATGACGCACAATCCCACTATCCTTCGCAAGACCCT<br>TCCTCTATATAAGGAAGTTCATTTCATTTGGAGAGGAC
rbcs promoter sequence	TGTGGGAACGAGATAAGGGCGAAGTGCGCTAGTAGCCTGCTATT<br>TAAAATATATCCACAATTTATAATGTATTTGAAGATTAGTCAATT<br>CGTCCAAAATTCAGGACTAAGTATCTTGAATTTTTGTATCCTGAA<br>TTTTGGGGCTACTAATTTGGAACTCAGGACTTAATGTCCTAAATT<br>TTTGAGCCGCTAATTTGAAATTCAGGACTAAGTGTTTTGAATTTTT<br>GAACTGCTTATTCGAAATGCAAGACTAAGTGACATGGATTTTTG<br>AACTGCTAATTTAATATTCAGGACATAAGATTCGAATTTTCAGAC<br>ATAATTTTTTAACTTTAGGGCACGATGTCCTGAAGTTTGAATCTT<br>GAGGTCTAAACTTCAAGATGCAGCGTCTTGAAGTTTAAGTGAAC<br>TGGCTAATCTTTAAATACTTGTAAACTGTGGATACATTTTTAAAT<br>AATATATTTAAAAGCGGCTACCTGGTATCATCTTCACGAGAATTT<br>TCCAAGTTAATTGTAAAGGAAATAGTGGTGTTGCATCAAGTTAT<br>GGACAATATAAGGAAGCAAACAGTACTCTAGCTATCAAATTAGT<br>TTCCACTTCTAAACCATGAATATTAGGAAAAACAAGAAACAAA<br>ACAAATATACATAAACAATACGGCTAAAGCCCAAGGAAAAGGG<br>ACTCTAAAAAAATTAACCAACCTCAATCACACATTCATATCCTC<br>TTCCTACCTCATCTAGGATGAGATAAGATTACTGAGGTGCTTACA<br>CGTGGCACCTCCATTGTGGTGACTAAATGAAGAGTGGCTTAGCT<br>CAAAATATAATTTTCCAACCTTTCATGTGTGGATATTAAGTTTTGT<br>GTAGTGAATCAAGAACCACATAATCCAATGGTTAGCTTTATTCC<br>AAGATGAGGGGGTTGTTGATTTTTGTCCGTCAGATATAGGAAAT<br>ATGTAAAACCTTATCATTATATATAGGGTGGTGGGCAACTATGC<br>AATGACCATATTGGAAGTTAAAGGAAAAGAGAGAAAGAGAAAT<br>CTTTCTGAATCGTCGACCTGCAGGCATG
TP sequence	TGGCTTCCTCTGTCATTTCTTCAGCAGCTGTTGCCACACGCAGCA<br>ATGTTACACAAGCTAGCATGGTTGCACCTTTCACTGGTCTCAAAT<br>CTTCAGCCACTTTCCCTGTTACAAAGAAGCAAAACCTTGACATC<br>ACTTCCATTGCTAGCAATGGTGGAAGAGTTAGCTGC
p1300BGFP	CGATCGGTCGATTCATAGAAGATTAGATTTTCATAGTATTTTTT

AAAGTAAACCTTTAACTACGGTTAGGACACTTTTAAGTTAAATTT
AATTTGAACCCTTAAATTAATTTTTAAAATAGATAAATATCAATC
ATCCTGATATGCTTTTGAAAAAATGAATGAGAAAGATGATTCAA
TTAAGGCCACATTTTAATCATGACTAAAATAATATACAGTATAAT
TTCATATATATTTGCTTTAAAAAAAAATTGACAATCCATTCGTTT
CTAGCAATAAATTTCTTCAACCACAAATATATTAAAGATAACTA
CGGCATAGAAACAAAAATCTATGAAGAATTTTTGTATACTTCAT
ATGAAATTAAAAAAAACTTCATTGAACATCAAAATAATAATAAT
AATCATAAACTCCTCAATATTTATATTCCTAGCTTCTTGAATTAA
ATTGTTTACATATTCAACGATGTAAAAAATTATTTCTCTATCTATT
TTCCTTATATCATGCATGGTTTCACATATATCAAAGGATAAAAGC
AATCTATGTAAATTATCTCACTTTATTAAGTTTTCTATCTGAATTA
TTGAGAACGTAGATTTCTTTTTGCACTATCCCCCAATAATTAGCA
AAACACACCTAGACTAGATTTGTTTTGCTAACCCAATTGATATTA
ATTATATATGATTAATATTTATATGTATATGGAATTGGTTAATAA
AATGCATCTGGTTCATCAAAGAATTATAAAGACACGTGACATTC
ATTTAGGATAAGAAATATGGATGATCTCTTTCTCTTATTCAGATA
ATTAGTAATTACACATAACACACAACTTTGATGCCCACATTATA
GTGATTAGCATGTCACTATGTGTGCATCCTTTTATTTCATACATTA
ATTAACTTGGCCAATCCAGAAGATGGACAAGTCTAGGGTCACAT
TGCAGGGTACTCTAGCTTACTCGCCTTCTTTTTCGAAGGTTTGAGT
ACCTTCAGGGCATCCTCTTGATACATTACTTTCCACTTCGATTGG
GGCAAGCTGTAGCAGTTCTTGCTTAGACCGAATTGCCATCTCACA
GAGATGCTGAAGAGTTCGCGACCCTCCAGAAACGGTGATACTAA
CTCCTCGAAACCGAATACTATAGGTACATCCGATCTGGTCGAAA
CCGAAAAATCGAGATGCTGCATAGTTAACCGAATCTCCCGTCCA
AGATCCAAGGACTCTGTGCAGTGAAGCTTCCGTCCTGTCGTATCT
GAGATATCTCTTAAATACAACTTTCCCGAAACCCCAGCTTTCCTT
GAAACCAAGGGGATTATCTTGATTCGAATTCGTCTCATCGTTATG
TAGCCGCCACTCAGTCCAACTCGGACTTTCGTCAGGAAGTTTGA
AGGGAGAAGTTGTACCTCCTGATCCTCCATCCCAACGTTCACTGT
TAGCTTGTTCCCTAGCGTCGTTTCCTTGTATAGCTCGTTCCATGGA
TTGTAAATAGTAATTGTAATGTTGTTTGTTGTTTGTTGTTGTTGGT
AATTGTTGTAAAAATACGCTCTCCAAATGAAATGAACTTCCTTAT
ATAGAGGAAGGGTCTTGCGAAGGATAGTGGGATTGTGCGTCATC
CCTTACGTCAGTGGAGATATCACATCAATCCACTTGCTTTGAAGA
CGTGGTTGGAACGTCTTCTTTTTCCACGATGCTCCTCGTGGGTGG
GGGTCCATCTTTGGGACCACTGTCGGCAGAGGCATCTTCAACGA
TGGCCTTTCCTTTATCGCAATGATGGCATTTGTAGGAGCCACCTT
CCTTTTCCACTATCTTCACAATAAAGTGACAGATAGCTGGGCAAT
GGAATCCGAGGAGGTTTCCGGATATTACCCTTTGTTGAAAAGTCT
CAATTGCCCTTTGGTCTTCTGAGACTGTATCTTTGATATTTTTGGA
GTAGACAAGTGTGTCGTGCTCCACCATGTTCTGGCAATTCCGGTT
CGCTTGCTGTCCATAAAACCGCCCAGTCTAGCTATCGCCATGTAA

GCCCACTGCAAGCTACCTGCTTTCTCTTTGCGCTTGCGTTTTCCCT
TGTCCAGATAGCCCAGTAGCTGACATTCATCCGGGGTCAGCACC
GTTTCTGCGGACTGGCTTTCTACGTGTTCCGCTTCCTTTAGCAGCC
CTTGCGCCCTGAGTGCTTGCGGCAGCGTGAAGCTGGCGCGCCGC
TCTAGCAGAAGGCATGTTGTTGTGACTCCGAGGGGTTGCCTCAA
ACTCTATCTTATAACCGGCGTGGAGGCATGGAGGCAAGGGCATT
TTGGTAATTTAAGTAGTTAGTGGAAAATGACGTCATTTACTTAAA
GACGAAGTCTTGCGACAAGGGGGGCCCACGCCGAATTTTAATAT
TACCGGCGTGGCCCCACCTTATCGCGAGTGCTTTAGCACGAGCG
GTCCAGATTTAAAGTAGAAAAGTTCCCGCCCACTAGGGTTAAAG
GTGTTCACACTATAAAAGCATATACGATGTGATGGTATTTGATGG
AGCGTATATTGTATCAGGTATTTCCGTCGGATACGAATTATTCGT
ACGACCCTCCTGCAGGcatacacctaaaaccgtgaacggtgtcggggaactcatttaaca
gtggatcccacaTTtTTcaaacgcatacttagcaacgaagtacttataataagcgggagaattatttA
agCTTTGAGCGCgtcatatgattggccgacaagagttaatggtagggcccacaaaaaaatcgc
gcggccatccggtaatattatacggatggccgcttttggAACCCAAAGGggaatttgaaTTT
CAAACaAacttctagttatttacagaatatgccatttggagacactataaattgtatccagtctccaat
tcccagagtaaaagcccatcagttgtctctaattgaccaaagtcaCTCGAGAAACAAACA
AAATCAACAAATATAGAAAATAACGCATTTCCAATTCTTTGAAA
TTTCTGCAACATCTAGAACAATGGTGAGCAAGGGCGAGGAGCTG
TTCACCGGGGTGGTGCCCATCCTGGTCGAGCTGGACGGCGACGT
AAACGGCCACAAGTTCAGCGTGTCCGGCGAGGGCGAGGGCGAT
GCCACCTACGGCAAGCTGACCCTGAAGTTCATCTGCACCACCGG
CAAGCTGCCCGTGCCCTGGCCCACCCTCGTGACCACCTTCAGCT
ACGGCGTGCAGTGCTTCAGCCGCTACCCCGACCACATGAAGCAG
CACGACTTCTTCAAGTCCGCCATGCCCGAAGGCTACGTCCAGGA
GCGCACCATCTTCTTCAAGGACGACGGCAACTACAAGACCCGCG
CCGAGGTGAAGTTCGAGGGCGACACCCTGGTGAACCGCATCGA
GCTGAAGGGCATCGACTTCAAGGAGGACGGCAACATCCTGGGG
CACAAGCTGGAGTACAACTACAACAGCCACAACGTCTATATCAT
GGCCGACAAGCAGAAGAACGGCATCAAGGTGAACTTCAAGATC
CGCCACAACATCGAGGACGGCAGCGTGCAGCTCGCCGACCACT
ACCAGCAGAACACCCCCATCGGCGACGGCCCCGTGCTGCTGCCC
GACAACCACTACCTGAGCACCCAGTCCGCCCTGAGCAAAGACC
CCAACGAGAAGCGCGATCACATGGTCCTGCTGGAGTTCGTGACC
GCCGCCGGGATCACTCACGGCATGGACGAGCTGTACAAGTAAG
AGCTCGAAGTGACATCACAAAGTTGAAGGTAATAAAGCCAAAT
TAATTAAGACATTTTCATAATGATGTCAAGAATGCAAAGCAAAT
TGCATAACTGCCTTTATGCAAAACATTAATATAATATAAATTATA
AAGAACTGCGCTCTCTGCTTCTTATTTTCTTAGCTTCATTTATTAG
TCACTAGCTGTTCAGAATTTTCAGTATCTTTTGATATTACTAAGA
ACCTAATCACACAATGTATATTCTTATGCAGGAAAAGCAGAATG
CTGAGCTAAAAGAAAGGCTTTTTCCATTTTCGAGAGACAATGAG
AAAAGAAGAAGAAGAAGAAGAAGAAGAAGAAGAAGAAGAAAAGA

GTAAATAATAAAGCCCCACAGGAGGCGAAGTTCTTGTAGCTCCA
TGTTATCTAAGTTATTGATATTGTTTGCCCTATATTTTATTTCTGTC
ATTGTGTATGTTTTGTTCAGTTTCGATCTCCTTGCAAAATGCAGAG
ATTATGAGATGAATAAACTAAGTTATATTATTATACGTGTTAATA
TTCTCCTCCTCTCTCTAGCTAGCCTTTTGTTTTCTCTTTTTCTTATTT
GATTTTCTTTAAATCAATCCATTTTAGGAGAGGGCCAGGGAGTG
ATCCAGCAAAACATGAAGATTAGAAGAAACTTCCCTCTTTTTTTT
CCTGAAAACAATTTAACGTCGAGATTTATCTCTTTTTGTAATGGA
ATCATTTCTACAGTTATGACGAATTGTACATCAACGAAAAATTA
GTCAAACGACTAAAATAAATAAATATCATGTGTTATTAAGAAAA
TTCTCCTATAAGAATATTTTAATAGATCATATGTTTGTAAAAAAA
ATTAATTTTTACTAACACATATATTTACTTATCAAAAATTTGACA
AAGTAAGATTAAAATAATATTCATCTAACAAAAAAAAAAACCAG
AAAATGCTGAAAACCCGGCAAAACCGAACCAATCCAAACCGAT
ATAGTTGGTTTGGTTTGATTTTGATATAAACCGAACCAACTCGGT
CCATTTGCACCCCTAATCATAATAGCTTTAATATTTCAAGATATT
ATTAAGTTAACGTTGTCAATATCCTGGAAATTTTGCAAAATGAAT
CAAGCCTATATGGCTGTAATATGAATTTAAAAGCAGCTCGATGT
GGTGGTAATATGTAATTTACTTGATTCTAAAAAAATATCCCAAGT
ATTAATAATTTCTGCTAGGAAGAAGGTTAGCTACGATTTACAGC
AAAGCCAGAATACAAAGAACCATAAAGTGATTGAAGCTCGAAA
TATACGAAGGAACAAATATTTTTAAAAAAATACGCAATGACTTG
GAACAAAAGAAAGTGATATATTTTTTGTTCTTAAACAAGCATCC
CCTCTAAAGAATGGCAGTTTTCCTTTGCATGTAACTATTATGCTC
CCTTCGTTACAAAAATTTTGGACTACTATTGGGAACTTCTTCTGA
AAATAGTGGTACCGAGTGTACTTCAAGTCAGTTGGAAATCAATA
AAATGATTATTTTATGAATATATTTCATTGTGCAAGTAGATAGAA
ATTACATATGTTACATAACACACGAAATAAACAAAAAAACACA
ATCCAAAACAAACACCCCAAACAAAATAACACTATATATATCCT
CGTATGAGGAGAGGCACGTTCAGTGACTCGACGATTCCCGAGCA
AAAAAAGTCTCCCCGTCACACATATAGTGGGTGACGCAATTATC
TTCAAAGTAATCCTTCTGTTGACTTGTCATTGATAACATCCAGTCT
TCGTCAGGATTGCAAAGAATTATAGAAGGGATCCCACCTTTTATT
TTCTTCTTTTTTCCATATTTAGGGTTGACAGTGAAATCAGACTGGC
AACCTATTAATTGCTTCCACAATGGGACGAACTTGAAGGGGATG
TCGTCGATGATATTATAGGTGGCGTGTTCATCGTAGTTGGTGAAG
TCGATGGTCCCGTTCCAGTAGTTGTGTCGCCCGAGACTTCTAGCC
CAGGTGGTCTTTCCGGTACGAGTTGGTCCGCAGATGTAGAGGCT
GGGGTGTCTGACCCCAGTCCTTCCCTCATCCTGGTTAGATCGGCC
ATCCACTCAAGGTCAGATTGTGCTTGATCGTAGGAGACAGGATG
TATGAAAGTGTAGGCATCGATGCTTACATGATATAGGTGCGTCTC
TCTCCAGTTGTGCAGATCTTCGTGGCAGCGGAGATCTGATTCTGT
GAAGGGCGACACGTACTGCTCAGGTTGTGGAGGAAATAATTTGT
TGGCTGAATATTCCAGCCATTGAAGCTTTGTTGCCCATTCATGAG

GGAACTCTTCTTTGATCATGTCAAGATACTCCTCCTTAGACGTTG
CAGTCTGGATAATAGTTCGCCATCGTGCGTCAGATTTGCGAGGA
GACACCTTATGATCTCGGAAATCTCCTCTGGTTTTAATATCTCCGT
CCTTTGATATGTAATCAAGGACTTGTTTAGAGTTTCTAGCTGGCT
GGATATTAGGGTGATTTCCTTCAAAATCGAAAAAAGAAGGATCC
CTAATACAAGGTTTTTTATCAAGCTGGATAAGAGCATGATAGTG
GGTAGTGCCATCTTGATGAAGCTCAGAAGCAACACCAAGGAAG
AAAATAAGAAAAGGTGTGAGTTTCTCCCAGAGAAACTGGAATA
AATCATCTCTTTGAGATGAGCACTTGGGGTAGGTAAGGAAAACA
TATTTAGATTGGAGTCTGAAGTTCTTGCTAGCAGAAGGCATGTTG
TTGTGACTCCGAGGGGTTGCCTCAAACTCTATCTTATAACCGGCG
TGGAGGCATGGAGGCAAGGGCATTTTGGTAATTTAAGTAGTTAG
TGGAAAATGACGTCATTTACTTAAAGACGAAGTCTTGCGACAAG
GGGGGCCCACGCCGAATTTTAATATTACCGGCGTGGCCCCACCT
TATCGCGAGTGCTTTAGCACGAGCGGTCCAGATTTAAAGTAGAA
AAGTTCCCGCCCACTAGGGTTAAAGGTGTTCACACTATAAAAGC
ATATACGATGTGATGGTATTTGATGGAGCGTATATTGTATCAGGT
ATTTCCGTCGGATACGAATTATTCGTACGGCCGGACCGGTCCCCT
AGGCCGGCCAATTCGAGATCGGCTGAGTGGCTCCTTCAATCGTT
GCGGTTCTGTCAGTTCCAAACGTAAAACGGCTTGTCCCGCGTCAT
CGGCGGGGGTCATAACGTGACTCCCTTAATTCTCCGCTCATGATC
AGATTGTCGTTTCCCGCCTTCAGTTTAAACTATCAGTATCAGTGTT
TGACAGGATATATTGGCGGGTAAACCTAAGAGAAAAGAGCGTTT
ATTAGAATAACGGATATTTAAAAGGGCGTGAAAAGGTTTATCCG
TTCGTCCATTTGTATGTGCATGCCAACCACAGGGTTCCCCTCGGG
ATCAAAGTACTTTGATCCAACCCCTCCGCTGCTATAGTGCAGTCG
GCTTCTGACGTTCAGTGCAGCCGTCTTCTGAAAACGACATGTCGC
ACAAGTCCTAAGTTACGCGACAGGCTGCCGCCCTGCCCTTTTCCT
GGCGTTTTCTTGTCGCGTGTTTTAGTCGCATAAAGTAGAATACTT
GCGACTAGAACCGGAGACATTACGCCATGAACAAGAGCGCCGC
CGCTGGCCTGCTGGGCTATGCCCGCGTCAGCACCGACGACCAGG
ACTTGACCAACCAACGGGCCGAACTGCACGCGGCCGGCTGCAC
CAAGCTGTTTTCCGAGAAGATCACCGGCACCAGGCGCGACCGCC
CGGAGCTGGCCAGGATGCTTGACCACCTACGCCCTGGCGACGTT
GTGACAGTGACCAGGCTAGACCGCCTGGCCCGCAGCACCCGCG
ACCTACTGGACATTGCCGAGCGCATCCAGGAGGCCGGCGCGGG
CCTGCGTAGCCTGGCAGAGCCGTGGGCCGACACCACCACGCCG
GCCGGCCGCATGGTGTTGACCGTGTTCGCCGGCATTGCCGAGTTC
GAGCGTTCCCTAATCATCGACCGCACCCGGAGCGGGCGCGAGG
CCGCCAAGGCCCGAGGCGTGAAGTTTGGCCCCCGCCCTACCCTC
ACCCCGGCACAGATCGCGCACGCCCGCGAGCTGATCGACCAGG
AAGGCCGCACCGTGAAAGAGGCGGCTGCACTGCTTGGCGTGCAT
CGCTCGACCCTGTACCGCGCACTTGAGCGCAGCGAGGAAGTGAC
GCCCACCGAGGCCAGGCGGCGCGGTGCCTTCCGTGAGGACGCAT

TGACCGAGGCCGACGCCCTGGCGGCCGCCGAGAATGAACGCCA
AGAGGAACAAGCATGAAACCGCACCAGGACGGCCAGGACGAA
CCGTTTTTCATTACCGAAGAGATCGAGGCGGAGATGATCGCGGC
CGGGTACGTGTTCGAGCCGCCCGCGCACGTCTCAACCGTGCGGC
TGCATGAAATCCTGGCCGGTTTGTCTGATGCCAAGCTGGCGGCCT
GGCCGGCCAGCTTGGCCGCTGAAGAAACCGAGCGCCGCCGTCT
AAAAAGGTGATGTGTATTTGAGTAAAACAGCTTGCGTCATGCGG
TCGCTGCGTATATGATGCGATGAGTAAATAAACAAATACGCAAG
GGGAACGCATGAAGGTTATCGCTGTACTTAACCAGAAAGGCGG
GTCAGGCAAGACGACCATCGCAACCCATCTAGCCCGCGCCCTGC
AACTCGCCGGGGCCGATGTTCTGTTAGTCGATTCCGATCCCCAGG
GCAGTGCCCGCGATTGGGCGGCCGTGCGGGAAGATCAACCGCT
AACCGTTGTCGGCATCGACCGCCCGACGATTGACCGCGACGTGA
AGGCCATCGGCCGGCGCGACTTCGTAGTGATCGACGGAGCGCCC
CAGGCGGCGGACTTGGCTGTGTCCGCGATCAAGGCAGCCGACTT
CGTGCTGATTCCGGTGCAGCCAAGCCCTTACGACATATGGGCCA
CCGCCGACCTGGTGGAGCTGGTTAAGCAGCGCATTGAGGTCACG
GATGGAAGGCTACAAGCGGCCTTTGTCGTGTCGCGGGCGATCAA
AGGCACGCGCATCGGCGGTGAGGTTGCCGAGGCGCTGGCCGGG
TACGAGCTGCCCATTCTTGAGTCCCGTATCACGCAGCGCGTGAG
CTACCCAGGCACTGCCGCCGCCGGCACAACCGTTCTTGAATCAG
AACCCGAGGGCGACGCTGCCCGCGAGGTCCAGGCGCTGGCCGC
TGAAATTAAATCAAAACTCATTTGAGTTAATGAGGTAAAGAGAA
AATGAGCAAAAGCACAAACACGCTAAGTGCCGGCCGTCCGAGC
GCACGCAGCAGCAAGGCTGCAACGTTGGCCAGCCTGGCAGACA
CGCCAGCCATGAAGCGGGTCAACTTTCAGTTGCCGGCGGAGGAT
CACACCAAGCTGAAGATGTACGCGGTACGCCAAGGCAAGACCA
TTACCGAGCTGCTATCTGAATACATCGCGCAGCTACCAGAGTAA
ATGAGCAAATGAATAAATGAGTAGATGAATTTTAGCGGCTAAAG
GAGGCGGCATGGAAAATCAAGAACAACCAGGCACCGACGCCGT
GGAATGCCCCATGTGTGGAGGAACGGGCGGTTGGCCAGGCGTA
AGCGGCTGGGTTGTCTGCCGGCCCTGCAATGGCACTGGAACCCC
CAAGCCCGAGGAATCGGCGTGAGCGGTCGCAAACCATCCGGCC
CGGTACAAATCGGCGCGGCGCTGGGTGATGACCTGGTGGAGAA
GTTGAAGGCCGCGCAGGCCGCCCAGCGGCAACGCATCGAGGCA
GAAGCACGCCCCGGTGAATCGTGGCAAGCGGCCGCTGATCGAA
TCCGCAAAGAATCCCGGCAACCGCCGGCAGCCGGTGCGCCGTC
GATTAGGAAGCCGCCCAAGGGCGACGAGCAACCAGATTTTTTCG
TTCCGATGCTCTATGACGTGGGCACCCGCGATAGTCGCAGCATC
ATGGACGTGGCCGTTTTCCGTCTGTCGAAGCGTGACCGACGAGC
TGGCGAGGTGATCCGCTACGAGCTTCCAGACGGGCACGTAGAG
GTTTCCGCAGGGCCGGCCGGCATGGCCAGTGTGTGGGATTACGA
CCTGGTACTGATGGCGGTTTCCCATCTAACCGAATCCATGAACC
GATACCGGGAAGGGAAGGGAGACAAGCCCGGCCGCGTGTTCCG

TCCACACGTTGCGGACGTACTCAAGTTCTGCCGGCGAGCCGATG
GCGGAAAGCAGAAAGACGACCTGGTAGAAACCTGCATTCGGTT
AAACACCACGCACGTTGCCATGCAGCGTACGAAGAAGGCCAAG
AACGGCCGCCTGGTGACGGTATCCGAGGGTGAAGCCTTGATTAG
CCGCTACAAGATCGTAAAGAGCGAAACCGGGCGGCCGGAGTAC
ATCGAGATCGAGCTAGCTGATTGGATGTACCGCGAGATCACAGA
AGGCAAGAACCCGGACGTGCTGACGGTTCACCCCGATTACTTTT
TGATCGATCCCGGCATCGGCCGTTTTCTCTACCGCCTGGCACGCC
GCGCCGCAGGCAAGGCAGAAGCCAGATGGTTGTTCAAGACGAT
CTACGAACGCAGTGGCAGCGCCGGAGAGTTCAAGAAGTTCTGTT
TCACCGTGCGCAAGCTGATCGGGTCAAATGACCTGCCGGAGTAC
GATTTGAAGGAGGAGGCGGGGCAGGCTGGCCCGATCCTAGTCAT
GCGCTACCGCAACCTGATCGAGGGCGAAGCATCCGCCGGTTCCT
AATGTACGGAGCAGATGCTAGGGCAAATTGCCCTAGCAGGGGA
AAAAGGTCGAAAAGGTCTCTTTCCTGTGGATAGCACGTACATTG
GGAACCCAAAGCCGTACATTGGGAACCGGAACCCGTACATTGG
GAACCCAAAGCCGTACATTGGGAACCGGTCACACATGTAAGTG
ACTGATATAAAAGAGAAAAAAGGCGATTTTTCCGCCTAAAACTC
TTTAAAACTTATTAAAACTCTTAAAACCCGCCTGGCCTGTGCATA
ACTGTCTGGCCAGCGCACAGCCGAAGAGCTGCAAAAAGCGCCT
ACCCTTCGGTCGCTGCGCTCCCTACGCCCCGCCGCTTCGCGTCGG
CCTATCGCGGCCGCTGGCCGCTCAAAAATGGCTGGCCTACGGCC
AGGCAATCTACCAGGGCGCGGACAAGCCGCGCCGTCGCCACTC
GACCGCCGGCGCCCACATCAAGGCACCCTGCCTCGCGCGTTTCG
GTGATGACGGTGAAAACCTCTGACACATGCAGCTCCCGGAGACG
GTCACAGCTTGTCTGTAAGCGGATGCCGGGAGCAGACAAGCCCG
TCAGGGCGCGTCAGCGGGTGTTGGCGGGTGTCGGGGCGCAGCCA
TGACCCAGTCACGTAGCGATAGCGGAGTGTATACTGGCTTAACT
ATGCGGCATCAGAGCAGATTGTACTGAGAGTGCACCATATGCGG
TGTGAAATACCGCACAGATGCGTAAGGAGAAAATACCGCATCA
GGCGCTCTTCCGCTTCCTCGCTCACTGACTCGCTGCGCTCGGTCG
TTCGGCTGCGGCGAGCGGTATCAGCTCACTCAAAGGCGGTAATA
CGGTTATCCACAGAATCAGGGGATAACGCAGGAAAGAACATGT
GAGCAAAAGGCCAGCAAAAGGCCAGGAACCGTAAAAAGGCCG
CGTTGCTGGCGTTTTTCCATAGGCTCCGCCCCCCTGACGAGCATC
ACAAAAATCGACGCTCAAGTCAGAGGTGGCGAAACCCGACAGG
ACTATAAAGATACCAGGCGTTTCCCCCTGGAAGCTCCCTCGTGC
GCTCTCCTGTTCCGACCCTGCCGCTTACCGGATACCTGTCCGCCT
TTCTCCCTTCGGGAAGCGTGGCGCTTTCTCATAGCTCACGCTGTA
GGTATCTCAGTTCGGTGTAGGTCGTTCGCTCCAAGCTGGGCTGTG
TGCACGAACCCCCCGTTCAGCCCGACCGCTGCGCCTTATCCGGT
AACTATCGTCTTGAGTCCAACCCGGTAAGACACGACTTATCGCC
ACTGGCAGCAGCCACTGGTAACAGGATTAGCAGAGCGAGGTAT
GTAGGCGGTGCTACAGAGTTCTTGAAGTGGTGGCCTAACTACGG

CTACACTAGAAGGACAGTATTTGGTATCTGCGCTCTGCTGAAGC
CAGTTACCTTCGGAAAAAGAGTTGGTAGCTCTTGATCCGGCAAA
CAAACCACCGCTGGTAGCGGTGGTTTTTTTGTTTGCAAGCAGCAG
ATTACGCGCAGAAAAAAAGGATCTCAAGAAGATCCTTTGATCTT
TTCTACGGGGTCTGACGCTCAGTGGAACGAAAACTCACGTTAAG
GGATTTTGGTCATGCATTCTAGGTACTAAAACAATTCATCCAGTA
AAATATAATATTTTATTTTCTCCCAATCAGGCTTGATCCCCAGTA
AGTCAAAAAATAGCTCGACATACTGTTCTTCCCCGATATCCTCCC
TGATCGACCGGACGCAGAAGGCAATGTCATACCACTTGTCCGCC
CTGCCGCTTCTCCCAAGATCAATAAAGCCACTTACTTTGCCATCT
TTCACAAAGATGTTGCTGTCTCCCAGGTCGCCGTGGGAAAAGAC
AAGTTCCTCTTCGGGCTTTTCCGTCTTTAAAAAATCATACAGCTC
GCGCGGATCTTTAAATGGAGTGTCTTCTTCCCAGTTTTCGCAATC
CACATCGGCCAGATCGTTATTCAGTAAGTAATCCAATTCGGCTA
AGCGGCTGTCTAAGCTATTCGTATAGGGACAATCCGATATGTCG
ATGGAGTGAAAGAGCCTGATGCACTCCGCATACAGCTCGATAAT
CTTTTCAGGGCTTTGTTCATCTTCATACTCTTCCGAGCAAAGGAC
GCCATCGGCCTCACTCATGAGCAGATTGCTCCAGCCATCATGCC
GTTCAAAGTGCAGGACCTTTGGAACAGGCAGCTTTCCTTCCAGC
CATAGCATCATGTCCTTTTCCCGTTCCACATCATAGGTGGTCCCTT
TATACCGGCTGTCCGTCATTTTTAAATATAGGTTTTCATTTTCTCC
CACCAGCTTATATACCTTAGCAGGAGACATTCCTTCCGTATCTTT
TACGCAGCGGTATTTTTCGATCAGTTTTTTCAATTCCGGTGATATT
CTCATTTTAGCCATTTATTATTTCCTTCCTCTTTTCTACAGTATTTA
AAGATACCCCAAGAAGCTAATTATAACAAGACGAACTCCAATT
CACTGTTCCTTGCATTCTAAAACCTTAAATACCAGAAAACAGCTT
TTTCAAAGTTGTTTTCAAAGTTGGCGTATAACATAGTATCGACGG
AGCCGATTTTGAAACCGCGGTGATCACAGGCAGCAACGCTCTGT
CATCGTTACAATCAACATGCTACCCTCCGCGAGATCATCCGTGTT
TCAAACCCGGCAGCTTAGTTGCCGTTCTTCCGAATAGCATCGGTA
ACATGAGCAAAGTCTGCCGCCTTACAACGGCTCTCCCGCTGACG
CCGTCCCGGACTGATGGGCTGCCTGTATCGAGTGGTGATTTTGTG
CCGAGCTGCCGGTCGGGGAGCTGTTGGCTGGCTGGTGGCAGGAT
ATATTGTGGTGTAAACAAATTGACGCTTAGACAACTTAATAACA
CATTGCGGACGT

Table S2. Codon frequency table of *Lactuca sativa*.

Codon	RSCU	Codon	RSCU	Codon	RSCU	Codon	RSCU
TTT	0.529	TTC	0.471	TTA	0.163	TTG	0.244
TCT	0.230	TCC	0.159	TCA	0.228	TCG	0.091
TAT	0.566	TAC	0.434	TAA	0.389	TAG	0.167
TGT	0.584	TGC	0.416	TGA	0.444	TGG	1.000

CTT	0.264	CTC	0.140	CTA	0.113	CTG	0.076
CCT	0.330	CCC	0.159	CCA	0.372	CCG	0.139
CAT	0.641	CAC	0.359	CAA	0.702	CAG	0.298
CGT	0.145	CGC	0.068	CGA	0.129	CGG	0.079
ATT	0.423	ATC	0.324	ATA	0.253	ATG	1.000
ACT	0.282	ACC	0.242	ACA	0.377	ACG	0.098
AAT	0.542	AAC	0.458	AAA	0.540	AAG	0.460
AGT	0.171	AGC	0.121	AGA	0.363	AGG	0.215
GTT	0.397	GTC	0.173	GTA	0.156	GTG	0.274
GCT	0.347	GCC	0.190	GCA	0.348	GCG	0.115
GAT	0.687	GAC	0.313	GAA	0.585	GAG	0.415
GGT	0.335	GGC	0.140	GGA	0.340	GGG	0.185

Table S3. Product testing in the p1300BR plasmid.

Original number	Test samples	Test samples	Lutein		β-Carotene		Zeaxanthin		β-cryptoxanthin		Lycopene		α-Carotene	
	Dilution volume（mL）	Sample quality（g）	Concentration（ng/mL）	Sample content（ng/g）	Concentration（ng/mL）	Sample content（ng/g）	Concentration（ng/mL）	Sample content（ng/g）	Concentration（ng/mL）	Sample content（ng/g）	Concentration（ng/mL）	Sample content（ng/g）	Concentration（ng/mL）	Sample content（ng/g）
Mock	0.500	0.510	3333.81	3267.160	39.91	39.112	3768.07	3692.738	94.25	92.366	40.61	39.798	41.33	40.504
p1300BR	0.500	0.511	193.6	189.284	4.14	4.048	6318.08	6177.239	17.66	17.266	4.53	4.429	7.57	7.401

Original number	Phytoene		Violaxanthin		Capsorubin		Capsanthin		Astaxanthin		Astaxanthin		Antheraxanthin	
	Concentration（ng/mL）	Sample content（ng/g）	Concentration（ng/mL）	Sample content（ng/g）	Concentration（ng/mL）	Sample content（ng/g）	Concentration（ng/mL）	Sample content（ng/g）	Concentration（ng/mL）	Sample content（ng/g）	Concentration（ng/mL）	Sample content（ng/g）	Concentration（ng/mL）	Sample content（ng/g）
Mock	8.88	8.702	1863.26	1826.009	102.37	100.323	1234.06	1209.388	724.74	710.251	648.530	635.564	455.539	446.432
p1300BR	0.98	0.958	359.83	351.809	251.47	245.864	19042.59	18618.097	741.51	724.980	663.663	648.869	286.326	279.943

Table S4. List of all primers used in this study.

Name	aim of use	Sequence (5′-3′)
crtF	35S:TP:CrtW (cloning site: *Pst*I+*Sac*I)	ATTCGTACGACCCTCCTGCAGTGAGACTTTTCAACAAAGGGTA
crtR		TTGTGATGTCACTTCGAGCTCTCATTAAGATTCACCTCTCCA
cZF	rbcs:TP:CrtZ (cloning site: *Pst*I+*Sac*I)	ATTCGTACGACCCTCCTGCAGTGTGGGAACGAGATAAGGGCG
cZR		TTGTGATGTCACTTCGAGCTC TCATTAAGCTCCTGAACTACT
L5F	qRT-PCR for Psy gene (GeneBank no:X68017.1)	TGGTGCAGGAGAACAGACGAACTT
L5R		AGCAGCATCGAGCATGTCAAATGG
L6F	qRT-PCR for CrtZ0 gene (GeneBank no:Y09225.1)	TCCCGTGCCATTTGTCTCCAGC
L6R		GCACAAAGCAAGCAGCCAACCT
L7F	qRT-PCR for Lcyb gene (GeneBank no:X86221.1)	AGTTGTTGGAATTGGTGGCACAGC
L7R		TTATGGCATTGGCAACGACAGGAG
L8F	qRT-PCR for Ccs gene (GeneBank no:X76165.1)	TTAGCACCCACATCAAAGCCAGAG
L8R		TGGTGAAGGGTCAACGCAACATAC
L9F	qRT-PCR for Ubi gene (GeneBank no:AY486137.1)	GAGCAGTGGAGTCCAGCATTAACC
L9R		TTCTGAGTCCAGCTACGAGCAGTG
L10F	qRT-PCR for CrtW gene	CTCCAGGAAGGCCAAGGCTTAATG
L10R		CAGTACCAGGTGCAGCATGATGAG
L11F	qRT-PCR for CrtW gene	GTTATGGTGGCAGTGGGACTTCATC
L11R		GCCTATGTGCTTGTATACGCCTTGT
